# Annual Report 2023

**DOI:** 10.3934/microbiol.2024004

**Published:** 2024-01-22

**Authors:** Xu Guo

**Affiliations:** Room 703, Luoke Times Center, Anli Road, Chaoyang District, Beijing 100101, China

## 1. Journal summary

*AIMS Microbiology* is an international Open Access journal devoted to publishing peer-reviewed, high quality, original papers in the field of microbiology. Together with the Editorial Office of *AIMS Microbiology*, I wish to testify my sincere gratitude to all authors, members of the editorial board and reviewers for their contribution to *AIMS Microbiology* in 2023.

In 2023, We received more than 240 manuscripts and 40 of them were accepted and published. These published papers include 22 research articles, 16 review articles, 1 editorial, and 1 Commentary. The authors of the manuscripts are from more than 20 countries. The data shows a significant increase of international collaborations on the research of microbiology.

An important part of our strategy has been preparation of special issues. 2 special issues published more than five papers. *AIMS Microbiology* have invited 18 new experts to join our Editorial Board in 2023. We will continue to renew Editorial Board in 2024.

In 2023, *AIMS Microbiology* received its first Impact Factor (4.8). In addition, based on the Scopus database of Elsevier, the CiteScore of *AIMS Microbiology* has been increased from 4.8 to 6.3.

We hope that in 2024, *AIMS Microbiology* can receive and collect more excellent articles to be able to publish. The journal will dedicate to publishing high quality papers by regular issues as well as special issues organized by the members of the editorial board. We believe that all these efforts will increase the impact and citations of the papers published by *AIMS Microbiology*.

On behalf of

*AIMS Microbiology Editorial Board*

## 2. Editorial development

### 2.1. Manuscript statistics

**Figure 1.**
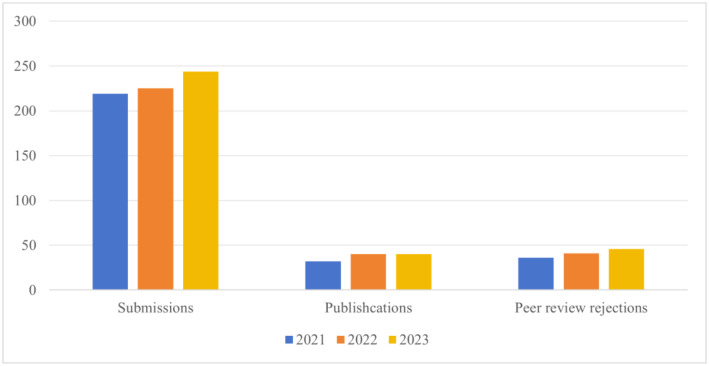
The number of manuscripts submitted, published, and rejected after peer review in the last 3 years.

The submissions of our *AIMS Microbiology* journal in 2023 increased (Figure 1). The journal received a total of 244 submissions, and 40 were online, with a peer review rejection rate of 53%, which shows that, despite the increase in submission, *AIMS Microbiology* has always maintained high standards and strict requirements. As shown in Table 1, these published papers include 22 research articles, 16 review articles, 1 editorial, and 1 Commentary. The publication time (from submission to online) was 95 days, illustrating the efficient review process. In 2023, *AIMS Microbiology* did not increase the number of publications. *AIMS Microbiology* places greater emphasis on publishing high-quality articles and hopes to receive more citations.

**Table 1.** The category of published articles.

Article type	Number
Research article	22
Review	16
Commentary	1
Editorial	1

### 2.2. Oline articles by country and region

**Figure 2.**
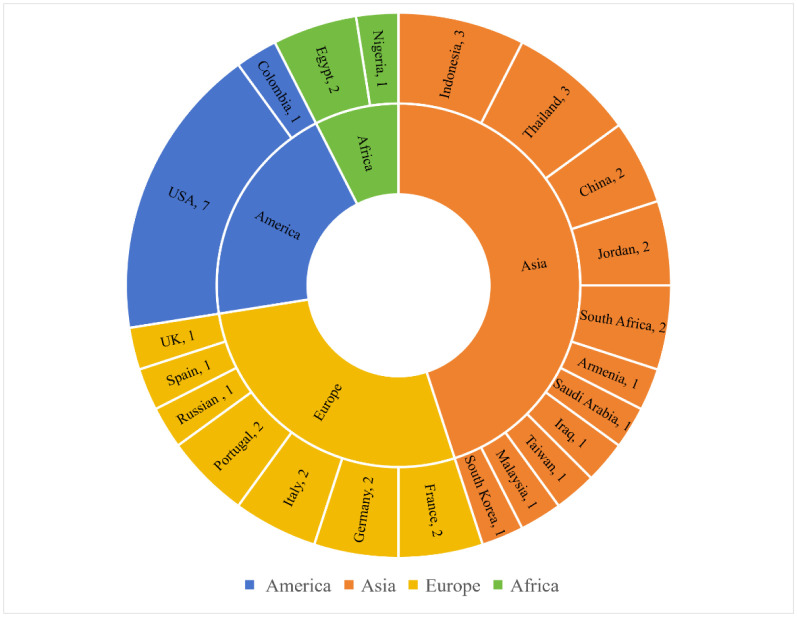
Oline articles by country and region.

Figure 2 provides the counts of online manuscripts per country and region. The country and region are derived by affiliation of the corresponding author. The authors of the online manuscripts come from more than 20 countries and regions, mainly from countries in Asia, the Americas and Europe, of which the most is the United States, with seven authors.

### 2.3. Special issues

In 2023, we set up six new special issues. 2 special issues and 1 topic issue published more than 5 manuscripts (Table 2). We encourage Editorial Board members to propose more potential topics, and to act as editors of special issues.

**Table 2.** Special issues and topic issues published more than 5 manuscripts in 2023.

Title	Papers
Biotechnological applications of microorganisms in Industry, Agriculture and Environment	10
Antimicrobials and Resistance	6
SARS-CoV-2 Antibodies and Immunity	5

### 2.4. Editorial Board members

**Figure 3.**
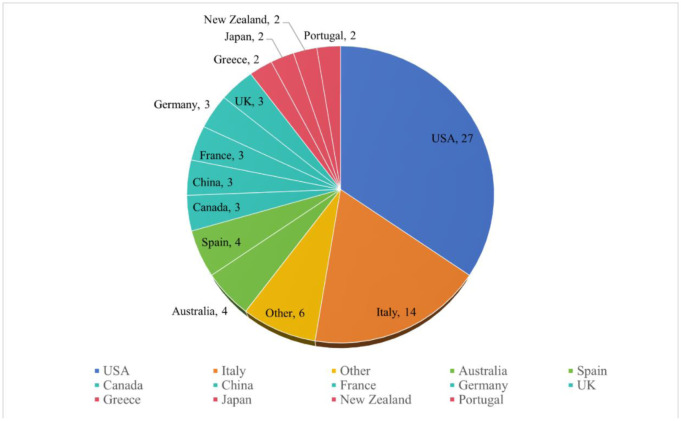
Country distribution of editorial board members.

*AIMS Microbiology* has Editorial Board members representing researchers from 20 countries, which are shown in Figure 3. We are constantly assembling the editorial board to be representative to a variety of disciplines across the field of microbiology. *AIMS Microbiology* has 80 members now, and 18 of them joined in 2023. Table 3 shows some new members of editorial board. We will continue to invite dedicated experts and researchers in order to renew the Editorial Board in 2024.

**Table 3.** Some new editorial board members

Name	Country	H-index
Mallavarapu Megharaj	Australia	81
Shih-Chin Cheng	China	20
Cinzia Randazzo	Italy	31
Gabriella Caruso	Italy	28
Fann Wu	USA	22

### 2.5. Citation impact

#### 2.5.1. CiteScore

**Figure 4.**
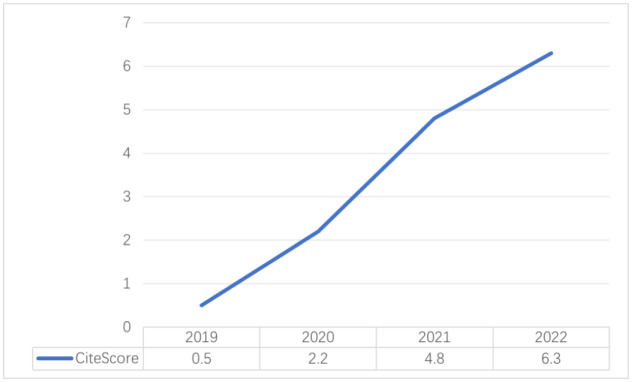
CiteScore in 2019–2022.

Calculating the CiteScore is based on the number of citations to documents (articles, reviews, conferencepapers, book chapters, and datapapers) by a journal over four years, divided by the number of the same document types indexed in Scopus and published in those same four years. As shown in Figure 4, the score of *AIMS Microbiology* has been increasing year by year.

#### 2.5.2. Impact Factor

Clarivate released the impact factor (IF) on June 28, 2023. *AIMS Microbiology* received it's first IF of 4.8. Five-year IF is 6.3.

#### 2.5.3. Highly Cited Papers in last 2 years

We counted citations for articles published in 2021 and 2022, and summarized 8 articles with more than 10 citations (Table 4).

**Table 4.** Highly cited papers in last 2 years.

No.	Article	Citations
1	Exploring endophytes for in *vitro* synthesis of bioactive compounds similar to metabolites produced in *vivo* by host plants	25
2	Biofilms: Formation, drug resistance and alternatives to conventional approaches	21
3	*Salmonella spp*. quorum sensing: an overview from environmental persistence to host cell invasion	20
4	Yeasts in different types of cheese	14
5	*Listeria monocytogenes* isolates from Western Cape, South Africa exhibit resistance to multiple antibiotics and contradicts certain global resistance patterns	13
6	Role of vitamin C in preventing of COVID-19 infection, progression and severity	12
7	Ways to improve biocides for metalworking fluid	12
8	Occurrence of plasmid mediated fluoroquinolone resistance genes amongst enteric bacteria isolated from human and animal sources in Delta State, Nigeria	10

### 2.6. Summary & plan

#### 2.6.1. Summary

We received more than 240 manuscript submissions and published 40 papers in 2023. The citation impact of our journal also obtained a great improve. Our biggest gain in 2023 was obtaining the first IF: 4.8. This IF exceeds the average level of all journal in microbiology. The CiteScore increased to 6.3.

#### 2.6.2. Plan in 2024

*AIMS Microbology* is devoted to publishing peer-reviewed, high quality, original papers in the field of microbiology. In 2024, we will make greater efforts to develop journal.

In 2024, we expect to publish more articles to enhance the reputation. We will invite more experts in different field of microbiology to publish an article. We will strive to attract Chinese experts to join our editorial board and also hope to gain more recognition and submissions from Chinese researchers. We encourage the editorial board to participate and lead the special issue.

## 3. Acknowledgments

We really appreciate the time and effort of all our Editorial Board Members and Guest Editors, as well as our reviewers devoted to our journal in such difficult circumstances. All your excellent professional effort and expertise provided us with very useful and professional suggestions in 2023. Last, but not least, thanks are given to the hard work of the in-house editorial team.

